# Genome-Wide Analysis of CPP Transcription Factor Family in Endangered Plant *Phoebe bournei* and Its Response to Adversity

**DOI:** 10.3390/plants14050803

**Published:** 2025-03-05

**Authors:** Ronglin Liu, Yizhuo Feng, Qingyan Li, Hua Wu, Shengzhou Guo, Junnan Li, Xiaomin Liu, Yanlin Zhang, Xinghao Tang, Shijiang Cao

**Affiliations:** 1 College of Forestry, Fujian Agriculture and Forestry University, Fuzhou 350002, China; d1315068727@163.com (R.L.); 15532106788@163.com (Y.F.); gsz19559162600@126.com (S.G.); 2 College of Jun Cao Science and Ecology (College of Carbon Neutrality), Fujian Agriculture and Forestry University, Fuzhou 350002, China; 15255665669@163.com (Q.L.); 17770899282@163.com (Y.Z.); 3 College of Food Science, Fujian Agriculture and Forestry University, Fuzhou 350002, China; w2918083657@163.com; 4 Fujian Academy of Forestry Sciences, Fuzhou 350012, China; ljnan009@foxmail.com; 5 State Key Laboratory of Tree Genetics and Breeding, College of Biological Sciences and Technology, Beijing Forestry University, Beijing 100083, China; liuxiaomin@bjfu.edu.com

**Keywords:** *CPP* gene family, *Phoebe bournei*, genome-wide identification, abiotic stress, expression analysis

## Abstract

The *CPP* gene family comprises transcription factor genes containing a conserved CRC domain, which is mainly involved in plant development and evolution. Although *CPP* genes have been widely studied in many plants, little is known about them in woody plants, especially in the endangered species *Phoebe bournei* (Hemsl.). In the genome of *Phoebe bournei,* we identified 11 *PbCPP* genes (*PbCPP1*-*PbCPP11*) distributed on four chromosomes, with large differences in the number of amino acids. They encode both acidic and alkaline proteins. A phylogenetic analysis showed that these *PbCPP* genes can be divided into three subfamilies, A, B, and C, which contain seven, two, and two genes, respectively. Through an interspecific collinearity analysis, we identified homologous *PbCPP* genes. A promoter cis-acting element analysis revealed that PbCPPs contain a variety of elements that respond to plant hormones, stress signals, and light and play a role in growth and development, and most *PbCPP* genes (except *PbCPP3* and *PbCPP8*) contain MYB binding site elements that regulate drought-induced stress responses, indicating that they play an important role in plant drought resistance. An expression analysis showed that *PbCPP3* and *PbCPP4* expression was high in the roots and stems and lower in the leaves, whereas the expression of most of the other genes was low in the roots, stems, and leaves. In addition, six representative *PbCPP* genes were detected using qRT-PCR. The results show significant differences in the expression of *PbCPP* genes under abiotic stress conditions (drought, cold, and salt), indicating that they play an important role in stress responses. This study preliminarily verified the role of the *PbCPP* gene family in different abiotic stress responses, which is of great significance for understanding its mechanism in plant growth and development and stress adaptation.

## 1. Introduction

Plants are inevitably challenged by various abiotic stressors throughout their life cycles [1,2]. These stress factors originate from the deviation in external environmental conditions from the range required for normal plant growth, seriously affecting the growth and development processes of plants [3]. As sessile organisms, plants have evolved sophisticated defense mechanisms involving three principal molecular strategies: (1) osmotic adjustment through compatible solutes, such as proline and glycine betaine, (2) reactive oxygen species detoxification via enzymatic systems (SOD, POD, CAT), and (3) transcriptional reprogramming mediated by ABA signaling and MAPK cascades [4,5,6,7]. These synergistic responses are controlled by a conserved regulatory network composed of *NAC*, *WRKY*, and *DREB* gene families. Although significant progress has been made in the study of stress response genes, systematic investigation of the *CPP* gene family in *Phoebe bournei* remains limited, particularly within subtropical forest ecosystems [8].

As a valuable native tree species endemic to southern China, *Phoebe bournei* occupies a critical ecological niche in subtropical montane forests (600–1200 m elevation) and is primarily distributed across the Fujian, Jiangxi, and Guangdong provinces. This IUCN Red List-assessed species demonstrates exceptional adaptability to nutrient-poor acidic soils while serving dual ecological roles: (1) as a keystone species maintaining forest structure through allelopathic root exudates and (2) as a carbon sequestration agent. Its high-density timber (0.72–0.85 g/cm^3^) and unique phytochemical profile further underscore its economic importance in sustainable forestry [9,10]. However, wild populations of this species have declined due to human activities and global climate change, and it has been listed as a national secondary protected plant [11]. Drought stress, cold stress, and salt stress pose serious threats to the survival and growth of *Phoebe bournei* [12,13,14,15]. Drought stress reduces the soil moisture content, making it difficult for the root system to absorb water, resulting in the closure of leaf stomata, weakened photosynthesis, and slowed growth, and prolonged drought may result in the death of the plant [16,17]. Cold stress affects the fluidity of cell membranes, enzyme activity, and physiological processes related to photosynthesis and respiration, which damages the cellular structure and function and inhibits the normal growth and development of the plant [18,19]. Cold stress affects cell membrane fluidity, enzyme activity, and physiological processes related to photosynthesis and respiration [20], impairing cell structure and function and inhibiting normal plant growth and development. Salt stress increases soil osmotic pressure, interfering with water and nutrient uptake by the root system and triggering ionic toxicity [21,22,23]. These abiotic stresses interact with each other and greatly affect the growth and ecological adaptability of *Phoebe bournei* [24,25,26,27]; therefore, it is extremely important to thoroughly investigate the role of the *CPP* gene family in the stress response of *Phoebe bournei* and explore its tolerance mechanisms.

The cysteine-rich polycomb protein (*CPP*) family of transcription factor genes belongs to a specific subfamily [28]. *CPPs* commonly possess the CXC structural domain, which is widespread in plants and animals but has not been found in yeast [29,30]. The CXC domain exhibits a conserved structure with a CXCX4CX3YCXCXCX6CX3CXCX2C sequence [31], and there is usually a linkage of variable length between two CXC motifs, which often contains conserved R-sequences (RNPXAFXPK) [32]. The region comprising the three conserved sequences is defined as the CRC structural domain, which is a key signature of the *CPP* transcription factor gene family [29]. In the plant domain, members of this family play an indispensable and important role in the developmental processes of reproductive tissues and in the regulation of cell division [33]. *CPP* transcription factors are deeply involved in a series of complex physiological processes, such as the regulation of plant growth and development, the response to various hormones, and stress responses, and their CXC domains are able to accurately regulate the expression of target genes by binding to specific DNA sequences. Because of the unique characteristics of the CXC domain, researchers have successfully identified members of the *CPP* transcription factor family in many plant species, such as *Arabidopsis thaliana* (L.) [18,20], *Oryza sativa* L. [33], *Cucumis sativus* L. [34], *Zea mays* L. [8], *Glycine max* (L.) [35], *Camellia sinensis* (L.) [36], *Solanum lycopersicum* L. [37], and others. For example, *TSO1* in *Arabidopsis thaliana*, as the first member of the *CPP* family to be identified and analyzed in depth, possesses a variety of functions, such as the regulation of cytoplasmic division, cell expansion, and floral tissue development [38,39], and *CPP* in *Triticum aestivum* L. is able to bind in vivo to a specific region within the in vitro and b1 tandem repeat sequences, a process that is a necessary prerequisite for the generation of mutations in *Triticum aestivum* [8]. At the same time, *CPP* family members also have significant tissue-specific expression characteristics [40]. For example, the expression levels of the *CsyCPP1* and *CsyCPP2* genes were the highest in the stems and the lowest in the flowers and mature leaves [36]. In *Cucumis sativus*, *CsCPP2* was highly expressed in the leaves; *CsCPP3* was mainly expressed in the leaves and fertilized ovaries; *CsCPP1* was highly expressed in unfertilized ovaries, with relatively low expression in the leaves, uninflated ovaries, and roots; and *CsCPP4* was mainly expressed in the ovaries at different developmental stages [34]. In *Camellia sinensis*, *CsCPP* is highly expressed mainly in young leaves and terminal buds in the active growth state [36].

In conclusion, as an important plant species, *Phoebe bournei* is also confronted with various stress factors in its living environment. Therefore, the study on the stress response of *CPP* genes in *Phoebe bournei* is of great significance. Firstly, the stress response of plants is directly related to their survival ability and adaptability in the natural environment. By gaining an in-depth understanding of the role of *CPP* genes in *Phoebe bournei* during the stress response, we can reveal the molecular mechanisms by which it copes with environmental stresses. Secondly, although the stress response of plants is closely related to the plant hormone response, the stress response places more emphasis on the direct response and adaptation process of plants to external environmental stresses. For example, research on *PtrCPP1* in poplar has found that it plays a crucial role in the differentiation of vascular tissues and the development of xylem [41]. When facing stresses such as drought, *CPP* genes may enhance the plant’s adaptability to adverse environments by coordinating the expression of cell wall synthesis genes. Therefore, focusing on the study of the stress response of *CPP* genes in *Phoebe bournei* can help provide new ideas and methods for breeding plant varieties with strong stress resistance.

The aim of this study was to comprehensively analyze the function and mechanism of action of the *CPP* gene family in *Phoebe bournei* in response to abiotic stress, as well as the interactions between *PbCPPs* and other genes [37]. We used bioinformatics methods to identify 11 *CPP* gene family members in the whole genome of *Phoebe bournei* and analyzed their gene structures (exon–intron, gene length, amino acid sequence), conserved domains, and promoter regions (focusing on stress-related cis-acting elements). The tissue-specific expression pattern of *CPP* genes was studied using transcriptome technology, and the expression dynamics of key genes under different stresses were verified using RT-qPCR. Our findings provide a solid foundation for the further exploration of the functions of the *CPP* gene family in *Phoebe bournei* under abiotic stresses.

## 2. Materials and Methods

### 2.1. Identification of PbCPP Gene Family Members and Analysis of Physicochemical Properties

In this study, we first obtained the AtCPP sequences, gff files, and other related files from the TAIR database (https://www.arabidopsis.org (accessed on 10 September 2024)) and downloaded the gff, nucleic acid sequences, and other files for *Phoebe bournei* from the Ensembl Plants database (https://plants.ensembl.org/index.html (accessed on 10 September 2024)). Subsequently, TBtools-IIv2.154 was used to accurately extract the CDS sequences from the genome annotation information of *Phoebe bournei*, simplify them, and translate them into protein files. Then, the CPP family protein sequences of *Arabidopsis thaliana* were compared with those of *Phoebe bournei*, and the annotation information was viewed in NCBI BLASTP (National Center for Biotechnology Information (https://www.ncbi.nlm.nih.gov/, accessed on 10 September 2024)) so as to preliminarily screen for the CPP family proteins of *Phoebe bournei*. The hidden Markov model (pfam03638) [42] of CPP conserved domains was downloaded from the InterPro database (https://www.ebi.ac.uk/interpro/ (accessed on 10 September 2024)), and the HMMER tool was used to preliminarily screen candidate members of the CPP family in *Phoebe bournei*. The protein sequences obtained using the above two methods were compared in detail, and the coinciding sequences were selected. The conserved domains were further examined using the SMART database (https://smart.embl.de/ (accessed on 10 September 2024)), InterPro database, and NCBI-CDD database (https://www.ncbi.nlm.nih.gov/Structure/bwrpsb/bwrpsb.cgi (accessed on 10 September 2024)), and the protein sequences without CPP conserved domains were eliminated [43]. TBtools was used to analyze the physical and chemical properties of CPP family proteins, and the WOLF PSORT website (https://wolfpsort.hgc.jp/ (accessed on 10 September 2024)) and Plant-mPLoc website (http://www.csbio.sjtu.edu.cn/bioinf/plant-multi/ (accessed on 10 September 2024)) were used to perform subcellular localization analysis, which laid a solid foundation for further exploration of the functions and characteristics of CPP family proteins in *Phoebe bournei*.

### 2.2. Chromosomal Localization of PbCPP Gene Family Members and Construction of Phylogenetic Tree

The chromosome distribution information for CPP family members was extracted from the gff file of *Phoebe bournei* using TBtools-IIv2.154, and a map of the genes’ chromosome distribution was created. The whole-genome protein sequences, gff files, and other related files of *Arabidopsis thaliana* and rice were derived from the TAIR10 database (http://www.arabidopsis.org/index.jsp (accessed on 11 September 2024)) and Rice Genome Annotation Project database (http://rice.plantbiology.msu.edu (accessed on 11 September 2024)) [32]. The method for identifying rice *CPP* genes is consistent with the method for identifying Phoebe bournei *CPP* genes. MEGA11.0 software was used to compare the CPP sequences of *Phoebe bournei*, *Arabidopsis thaliana,* and rice. The parameters were set to their default values. ClustalW was used to compare the sequences, and the adjacency method was used. The Poisson model was used to perform 1000 self-expanding replicates to ensure the reliability of the results [44]. The original image of the constructed phylogenetic tree was beautified on the iTOL website (https://itol.embl.de/ (accessed on 11 September 2024)).

### 2.3. Tissue Expression Profile Analysis of PbCPP Genes

The sequence number of *Phoebe bournei*, PRNA628065, was searched on the European Bioinformatics Centre’s repository EBI (accessed on 7 September 2024). The transcriptome expression data in five different tissues of *Phoebe bournei* were downloaded [45]. An RNA-seq analysis was performed on these data to study the differential expression of 11 *PbCPP* genes. TBtools-IIv2.154 was used to convert the FPKM value algebra in the analysis of the data into log2 values, which were then used to generate a heat map.

### 2.4. PbCPP Gene Family Motifs, Structural Domains, and Gene Structure Analysis

The gene structure of the *CPP* family was extracted from the genome annotation file for *Phoebe bournei* using TBtools-IIv2.154 and visualized. The 11 identified *PbCPP* sequences were put into MEME (https://meme-suite.org/meme/tools/meme (accessed on 11 September 2024)) with the parameters set to default values to identify the conserved motifs [46,47]. The identified PbCPP sequences were uploaded to the online NCBI BLASTP (https://www.ncbi.nlm.nih.gov (accessed on 11 September 2024)) to display the intron–exon gene structure [45]. Finally, TBtools-IIv2.154 was used for visualization.

### 2.5. The Analysis of Cis-Acting Elements of the PbCPP Gene Family

The promoters of the *CPP* genes in *Phoebe bournei* were extracted using TBtools-IIv2.154, with the 2000 bp upstream sequence set as the gene transcription initiation site. The extracted promoter sequences were submitted to the PlantCARE website (https://bioinformatics.psb.ugent.be/webtools/plantcare/html/ (accessed on 11 September 2024)), and the output table data were screened and processed. The start and end positions of the promoter were determined, and finally, the results were visualized with the function in TBtools-IIv2.154.

### 2.6. Intraspecies and Interspecies Covariance Analysis of the PbCPP Gene Family

In order to analyze the relationship between the *CPP* gene family members of *Phoebe bournei* and those of other species (*Solanum lycopersicum*, *Oryza sativa*, *Cucumis sativus*, *Arabidopsis thaliana,* and *Triticum aestivum*), their gff files and gene proteomes were retrieved from Ensembl Plants (https://plants.ensembl.org/index.html (accessed on 11 September 2024)), and the relationships were analyzed and visualized using TBtools. In order to obtain the intraspecific collinearity of *PbCPPs*, the TBtools-IIv2.154 program was used for analysis and visualization [48], and the PS2024 software was used for beautification.

### 2.7. Plant Materials and Abiotic Stress Treatments

The material used in this experiment was “*Phoebe bournei*” (germplasm number: Jian’ou No.8). Phenotypic characteristics: The bark is grayish white or yellowish brown, the young branches are cylindrical, and the surface is smooth. The leaves are leathery or thick and leathery, lanceolate or inverted lanceolate, and smooth and shiny; the backs of the leaves are pubescent, and the veins are obvious, forming a dense grid, as observed in a one-year-old tree seedling purchased from the Fujian Academy of Forestry Sciences. It was cultivated under natural conditions and treated with drought (10% PEG6000), salt, and cold stress. During salt and cold stress, five time points were set: 2 h, 6 h, 12 h, 24 h, and 0 h (control group). During drought treatment, five time points were set: 0 h, 4 h, 8 h, 12 h, and 24 h. Each treatment was repeated three times, and mature leaves were sampled within a set time. After treatment, *Phoebe bournei* leaves were immediately stored in liquid nitrogen at −80 °C for future RNA extraction.

### 2.8. RNA Extraction and RT-qPCR Analysis

Total RNA was extracted from control and stress-treated samples using Omega Bio-Tek’s (Shanghai, China) RNA extraction kit. In strict compliance with the manufacturer’s guidelines, 5 μg of total RNA, 1 μL of gene-specific primers (10 μM), 4 μL of 5 × RT Mix (containing dNTPs and buffer), and 1 μL of M-MLV 4 reverse transcriptase were added to the 20 μL reaction system, and the volume was supplemented with RNase-free H_2_O. The reaction system was incubated at 50 °C for 30 min to complete first-strand cDNA synthesis, followed by heating at 85 °C for 15 min to inactivate the reverse transcriptase. The synthesized cDNA was diluted 5-fold with nuclease-free water, quantified via a NanoDrop 2000 spectrophotometer (Thermo Fisher Scientific, Waltham, MA, USA) with A260/A280 ratios of 1.8–2.0, and adjusted to a final working concentration of 50 ng/μL for qPCR analysis. Quantitative RT-PCR was performed using TransStart Top Green qPCR SuperMix (Transgen, Beijing, China). The reaction mixture consisted of 1 µL of cDNA, 2 µL of specific primers, 10 µL of SYBR Premix Ex TaqTM II, and 7 µL of ddH_2_O. The qRT-PCR reaction process was as follows: degeneration at 95 °C for 30 s, then 40 cycles of denaturation at 95 °C for 5 s, 60 °C for 30 s, 95 °C for 5 s, 60 °C for 60 s, and 50 °C for 30 s. The qRT-PCR experiment utilized specific primers (Table S1), which were designed through the Primer 3 website (http://bioinfo.ut.ee/primer3-0.4.0/ (accessed on 7 August 2024). PbEF1α (GenBank number, KX682032.1) was selected as the reference gene [49]. The relative expression of the *PbCPP* genes was calculated using the delta–delta Ct method, and one-way ANOVA with a confidence level of 95% and Duncan multiple comparison tests were performed using GraphPad Prism9.0 software (https://www.graphpad.com/ (accessed on 20 September 2024)) [50,51]. To ensure robustness, all quantitative PCRs were conducted with three biological replicates and three technical replicates.

## 3. Results

### 3.1. Identification of PbCPP Gene Family and Analysis of Physicochemical Properties

Through the view of NCBI BLASTP annotation information and the screening of the Pfam number in HMMER, with the help of Tbtools, NCBI-CDD, etc., we identified and screened the CPP conserved structural domains and finally obtained 11 *PbCPP* family members, *PbCPP1*-*PbCPP11* (Table 1). The number of amino acids in the 11 *CPP* genes ranged from 142 to 813, with *PbCPP4* having the most and *PbCPP1* having the fewest. The molecular weights of the proteins ranged from 15,718.6 to 88,999.2 Da, with *PbCPP4* being the largest and *PbCPP1* the smallest. The isoelectric points ranged from 5.46 to 8.92: they were greater than 7 for *PbCPP5*, *PbCPP6*, *PbCPP7*, *PbCPP8*, *PbCPP9,* and *PbCPP10* and less than 7 for the rest, indicating that there are both acidic and basic proteins in this family. The instability indices ranged from 41.51 to 72.08, with *PbCPP2* having the largest and *PbCPP6* the smallest. The lipolysis index ranged from 51.65 to 68.99, with *PbCPP11* being the largest and *PbCPP2* the smallest. The average coefficients of hydrophilicity ranged from −0.864 to −0.445, with *PbCPP10* being the smallest and *PbCPP7* being the largest, indicating that the *PbCPPs* have high hydrophilicity. The results of subcellular localization show that all members of the *PbCPP* family are localized in the nucleus.

### 3.2. Chromosomal Localization Analysis of the PbCPP Gene Family

**Figure 1:**
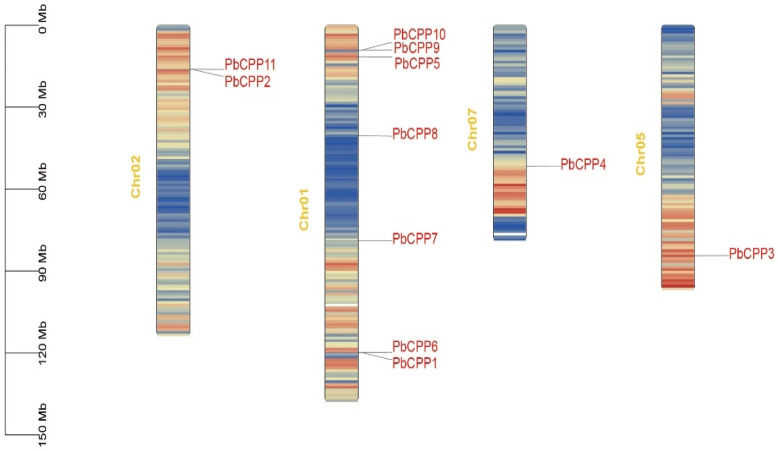
The identified positions of *PbCPP* genes on chromosomes in *Phoebe bournei*. The chromosomal locations of 11 mapped *PbCPP* genes are displayed. The scale unit is Mb. The chromosome number is marked on the left side of the corresponding chromosome. chr: chromosome.

In order to determine the chromosomal locations of *PbCPP* gene family members in *Phoebe bournei*, we used the genome annotation file and TBtools software (Figure 1). The mapping results show that the 11 identified *PbCPP* genes are distributed on four chromosomes: Chr01, Chr02, Chr05, and Chr07. Specifically, *PbCPP2* and *PbCPP11* are located on Chr02, *PbCPP4* is located on Chr07, *PbCPP3* is located on Chr05, and *PbCPP1*, *PbCPP5*, *PbCPP6*, *PbCPP7*, *PbCPP8*, *PbCPP9*, and *PbCPP10* are located on Chr01. *PbCPP1* and *PbCPP6*, *PbCPP9* and *PbCPP10* were tightly packed into a cluster on chr02, respectively. *PbCPP2* and *PbCPP11* were tightly packed into a cluster on chr01.

### 3.3. Analysis of Tissue-Specific Expression Patterns of PbCPP Gene Family

**Figure 2:**
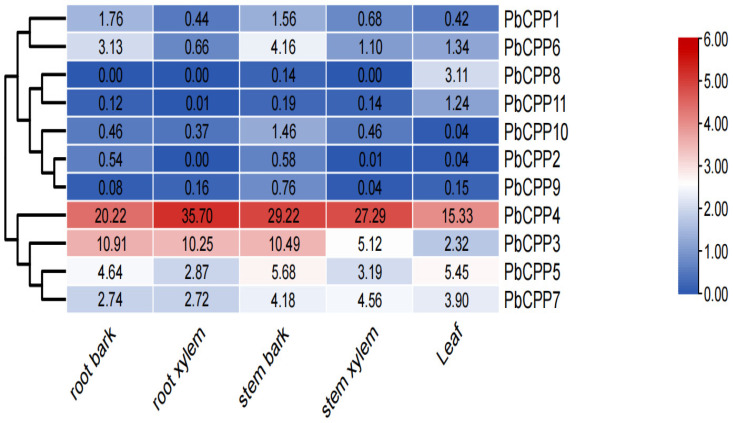
Tissue-specific gene expression patterns of 11 *PbCPP* genes. Expression patterns of genes in root xylem, root bark, stem xylem, stem bark, and leaves. Red and blue colors indicate high and low transcript abundances, respectively.

The tissue-specific expression pattern analysis of *PbCPPs* (Figure 2) showed that the expression patterns of *PbCPPs* were different in different genes and tissues. *PbCPP3* and *PbCPP4* were highly expressed in the roots and stems of *Phoebe bournei*, while their expression levels in leaves were relatively low. In contrast, *PbCPP1*, *PbCPP2*, *PbCPP8*, *PbCPP9*, *PbCPP10*, and *PbCPP11* had low or no expression in all tissues, and some genes, such as *PbCPP8*, were not expressed in specific tissues (e.g., roots). These results indicate that *PbCPP* genes have obvious tissue specificity. *PbCPP3* and *PbCPP4* are preferentially expressed in the roots and stems, while genes such as *PbCPP2* and *PbCPP8* have limited expression in all tissues. Therefore, *PbCPP3* and *PbCPP4* may play an important role in the growth and development of *Phoebe bournei* roots and stems.

### 3.4. Phylogenetic Tree and Cis-Acting Element Analysis

**Figure 3:**
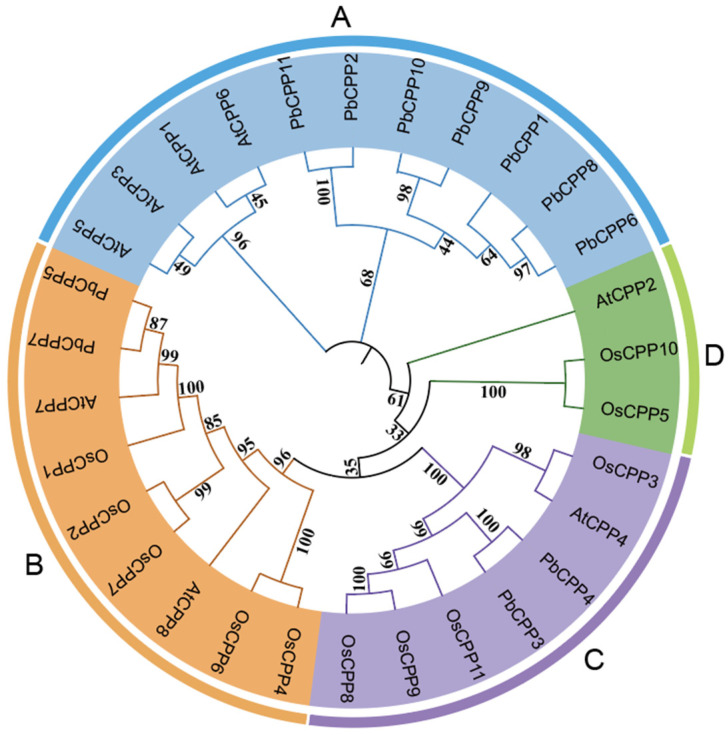
Evolutionary analysis of the *PbCPP* gene family. Note: *PbCPP* is a CPP in *Phoebe bournei*, *OsCPP* is a CPP in rice, and *AtCPP* is a CPP in *Arabidopsis thaliana*. Lines with different colors represent subfamilies of the CPP family. MEGA11 was used for 1000 replicates. The tree was constructed from 11 *PbCPPs* identified in *Phoebe bournei*, 8 *AtCPPs* identified in *Arabidopsis thaliana,* and 11 *OsCPPs* identified in rice.

A phylogenetic tree shows the affinity and evolutionary relationship between species and genes. In order to study the evolutionary relationship between the *PbCPP* gene family and other species, we used multiple sequence comparisons of *Arabidopsis thaliana*, rice (*Oryza sativa*), and *Phoebe bournei* to construct a phylogenetic tree of *PbCPPs* (Figure 3). *Arabidopsis thaliana*, *Phoebe bournei,* and rice have 8, 11, and 11 CPP members, respectively. These genes were rearranged into four subfamilies (A–D). *PbCPP* genes were unevenly distributed among different components. There are seven members in the A subfamily (*PbCPP1*, *PbCPP2*, *PbCPP6*, *PbCPP8*, *PbCPP9*, *PbCPP10*, *PbCPP11*). The B and C subfamilies contain two *PbCPP* members. A further analysis revealed that *PbCPP3* and *PbCPP4* in the C group are closely related to *OsCPP8*, *OsCPP9,* and *OsCPP11*. In group D, *OsCPP11* is closely related to *PbCPP5*, *PbCPP7,* and *AtCPP7*, indicating that *Phoebe bournei* is closely related to *Arabidopsis thaliana* and *Oryza sativa*; therefore, dicotyledonous and monocotyledonous plants may evolve in different directions after differentiation. The evolutionary origins of *PbCPP1*, *PbCPP6, PbCPP8*, *PbCPP9*, and *PbCPP10* all come from the same small branch, indicating their evolutionary homology. From the phylogenetic tree, we can obtain the relationship between *CPP* genes in different species, which is convenient for further study on the function of *CPP* genes in *Phoebe bournei*.

### 3.5. Analysis of Promoter Cis-Acting Elements of the PbCPP Gene Family

**Figure 4:**
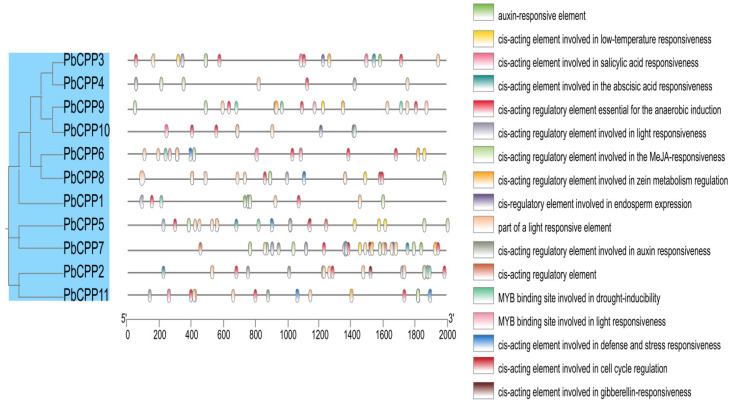
Analysis of cis-acting elements of promoter genes. Note: Cis-element prediction for the 11 *PbCPP* gene promoter sequences (−2000 bp) was performed using PlantCARE technology. The figure shows 17 types of cis-elements.

In order to understand the biological functions and regulatory network of *PbCPP* genes, we examined the cis-regulatory elements in the 2000 bp upstream promoter regions of *PbCPP* genes. Data processing and screening resulted in 17 functional groups (Figure 4). Based on their functions, we could broadly classify these cis-acting elements into four categories: hormone-responsive elements, stress-signaling elements, light-responsive elements, and growth-and-development-related elements. In the promoter regions of *PbCPP* family members, we found a large number of light-responsive elements, abscisic acid-induced responsive elements, hypoxia-induced elements, drought-induced elements, etc., which are closely related to the processes of plant growth and development, hormone induction, and abiotic stress induction. Among them, the light-responsive element is the most abundant cis-acting element in the promoter regions of *PbCPP* genes and is present in most members of the *PbCPP* gene family. In addition, we found that *PbCPP1*, *PbCPP2*, *PbCPP5*, *PbCPP6*, *PbCPP9*, and *PbCPP10* contain cis-elements that regulate drought-induced MYB binding sites, suggesting that the *PbCPP* gene family plays an important role in regulating plant resistance to drought stress. In addition, we found that *PbCPP3*, *PbCPP5*, *PbCPP6*, *PbCPP7*, *PbCPP8*, *PbCPP9*, and *PbCPP11* contain cis-elements regulating cold stress responsiveness, which suggests that the *PbCPP* gene family plays an important role in regulating plant resistance to cold stress. Statistical results show that, on average, each *PbCPP* gene contains about 30 cis-acting elements. Among them, cis-regulatory elements essential for anaerobic induction are present in all *PbCPP* genes. However, cis-acting elements associated with hormone responsiveness, stress signaling, light responses, and growth and development are relatively few in number in *PbCPP4*, with only 10. In addition, cis-acting elements involved in defense and stress responses are present only in some of the *PbCPP* genes. These findings indicate that the functional expression of *PbCPP* genes is complexly regulated by multiple cis-acting elements, which are closely related to plant developmental processes, hormonal responses, and abiotic stress responses.

### 3.6. Analysis of Covariance Between Phoebe Bournei and Different Species

**Figure 5:**
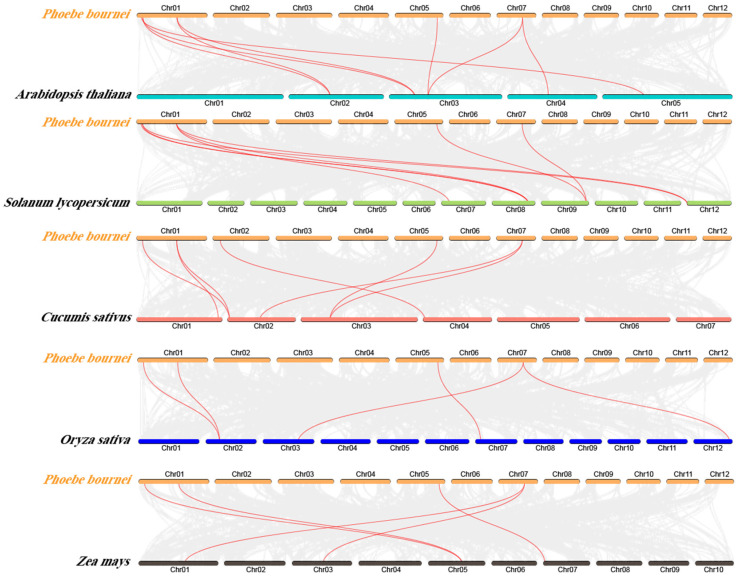
Covariance analysis of *PbCPP* genes with five representative plant species. Gray lines in the figure represent covariance regions between the genomes of Betula *Phoebe bournei* and other plants, while red lines highlight covariant *PbCPP* gene pairs.

In order to further understand the evolutionary process of the *CPP* gene family, we also analyzed the interspecies collinearity of the gene family (Figure 5). *Arabidopsis Thaliana* (model plant), *Oryza sativa*, *Solanum lycopersicum*, *Cucumis sativus,* and *Zea mays* were studied for interspecific CPP analysis to check the consistency of their gene sequences. The results show that *PbCPPs* form eight, five, eight, seven, and five collinear gene pairs with *AtCPP*, *OsCPP*, *SlCPP*, *CsCPP*, and *ZmCPP*, respectively. Multiple *PbCPP* genes were identified as homologous to single *AtCPP*, *OsCPP*, *SlCPP*, *CsCPP*, and *ZmCPP* genes. Similarly, there are multiple *AtCPP*, *OsCPP*, *SlCPP*, *CsCPP*, and *ZmCPP* genes derived from a single *PbCPP* gene. In *Phoebe bournei* from southern Fujian, the genes that homologous relationship with cucumber are located on chromosomes Chr01, Chr02, Chr05 and Chr07 in southern Fujian, and the genes that established homologous relationships with the remaining four plants are located on chromosomes Chr01, Chr05, and Chr07 in southern Fujian. A total of 33 collinear gene pairs were found in these five species, indicating that they have played conserved roles in evolution.

### 3.7. Analysis of PbCPP Gene Family Covariance

**Figure 6:**
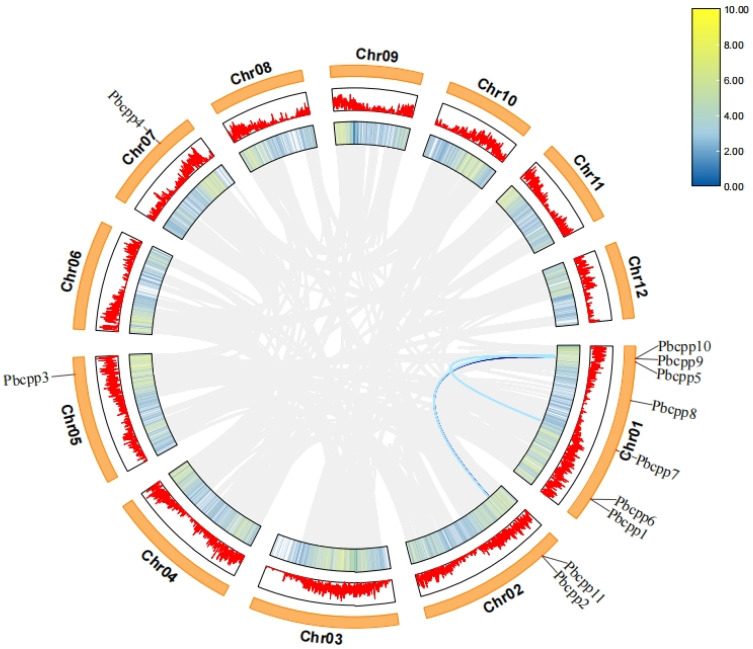
The genome map of *Phoebe bournei* is displayed as a circle. The outer segments of the circle correspond to the 12 assembled chromosomes, which are labeled sequentially from chromosome 1 (Chr01) to chromosome 12 (Chr12). Looking inward from the outermost edge of each chromosome segment, the first circle presents nucleotide positions measured in megabases (Mb), which scale the genetic map. Immediately adjacent to this is a visual display of gene density, where the peaked portions imply regions of denser genes. The gray lines in the innermost circle represent all replicated gene pairs in the *Phoebe bournei* genome, while the blue lines indicate the co-located gene pairs of PbCPP.

*Phoebe bournei* has 12 chromosomes. The intraspecific collinearity analysis of *PbCPP* genes revealed that 10 are located on chromosome 1 or 2 (Figure 6). Among the chromosomes containing *PbCPP* genes, chromosome 1 has the most *PbCPP* gene members at seven (0.64%), specifically *PbCPP1*, *PbCPP5*, *PbCPP6*, *PbCPP7*, *PbCPP8*, *PbCPP9*, and PbCPP10. However, there is only one *PbCPP* gene each on chromosomes 5 (*PbCPP3*) and 7 (*PbCPP4*). The non-uniform distribution pattern of *PbCPP* genes on chromosomes may be the result of genetic variation during evolution. In addition, three pairs of repetitive event fragments involving five *PbCPP* genes were successfully explored. It is particularly noteworthy that *PbCPP2*, *PbCPP5*, and *PbCPP7* on different chromosomes are collinearly associated, and *PbCPP10* and *PbCPP11* are also identified as homologous. The final results of collinearity analysis fully demonstrate the functional diversity of CPP transcription factors in *Phoebe bournei*.

### 3.8. Analysis of PbCPP Gene Family Motifs, Structural Domains, and Gene Structures

**Figure 7:**
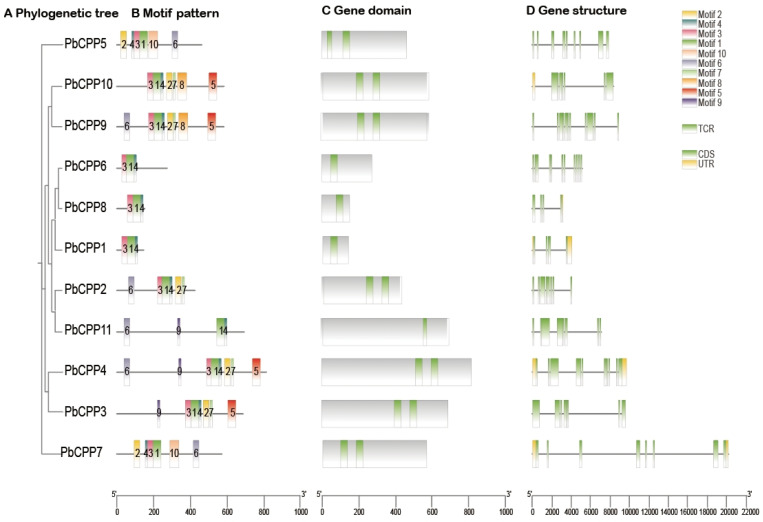
(**A**) The phylogenetic tree of *PbCPPs*. (**B**) The motifs of *PbCPPs*. Motifs 1–10 are displayed in rectangles with different colors. The bottom scale can be used to estimate the protein length. (**C**) PbCPPs with conserved domains. (**D**) The gene structures of *PbCPPs*. The green boxes represent exons (coding sequence, CDS), the black lines represent introns, and the yellow boxes represent the 5′ and 3′ untranslated regions.

An analysis of the exon–intron organization of the 11 *PbCPP* genes showed that the number of exons in these genes ranges from 4 (*PbCPP1* and *PbCPP8*) to 11 (*PbCPP6* and *PbCPP9*). The results also show that all 11 genes contain exons of varying lengths and that 55% of the PbCPPs have no introns (Figure 7D). The structural features of the *PbCPP* genes are comparable within the same subfamily but differ between subfamilies. The distribution of conserved protein motifs is the same for *PbCPP5* and *PbCPP7*; the same for *PbCPP1*, *PbCPP6*, and *PbCPP8*; The distribution of protein conserved motifs was essentially the same for *PbCPP4* and *PbCPP3* (Figure 7B); essentially the same for *PbCPP9* and *PbCPP1*, indicating that these genes express proteins with the same function, members of the same subfamily have the same motif composition and order. *PbCPP9* and *PbCPP10* both contain motifs 1, 2, 3, 4, 5, 7, and 8, and *PbCPP1*, *PbCPP6*, and *PbCPP8* all contain motifs 1, 3, and 4, which suggests that they have a similar function. All genes except *PbCPP11* contain motif 3, suggesting that motif 3 is relatively well conserved. Furthermore, analysis of the conserved structural domains of all genes in the four subfamilies revealed similarities. It is also worth noting that exon–intron structure analysis indicated that, among the 11 genes, only *PbCPP1*, *PbCPP4*, *PbCPP7*, *PbCPP8*, and *PbCPP10* have UTRs (untranslated regions) (Figure 7), which are located on both sides of the coding sequences of the genes. and do not encode amino acids but play a crucial role in the binding to the ribosomes and in the recognition of the initiation of the coding process. Although this region does not encode amino acids, it strongly affects translation efficiency because of its critical role in binding to the ribosome and recognizing the start of the coding process.

Although this region does not encode amino acids, it strongly affects translation efficiency because of its critical role in binding to the ribosome and recognizing the start site.

### 3.9. Expression Profiles of PbCPP Gene Family Under Cold Stress, Drought, and Salt Stress

**Figure 8:**
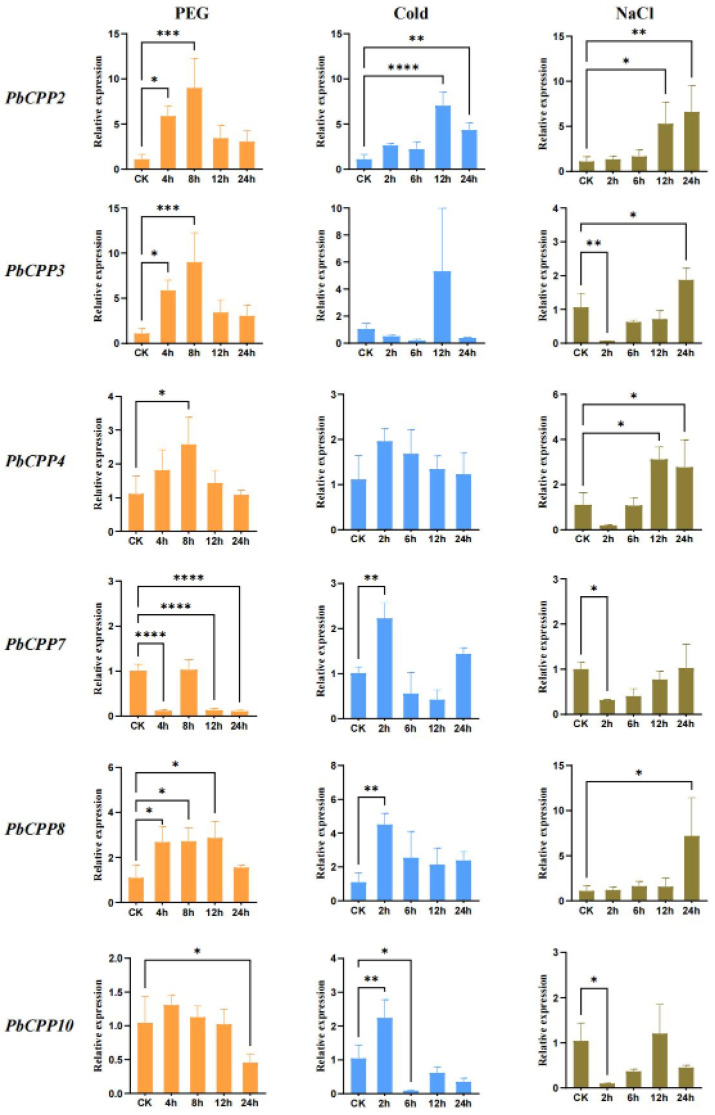
Expression profiles of *PbCPP* genes under various stresses. Error bars indicate standard deviations (SDs). The statistical analysis employed one-way ANOVA to discern significant differences, with the number of ‘*’s representing the level of significant differences (* *p* ≤ 0.05; ** *p* ≤ 0.005; *** *p* ≤ 0.0005; **** *p* ≤ 0.0001).

In order to study the responses of *PbCPP* genes in different stress environments (drought stress, cold stress, salt stress), six genes from four subfamilies (A–D) were selected for analysis under three different stress conditions. These six *PbCPP* genes are located in different subfamilies. Moreover, through experimental methods such as transcriptome analysis, it has been found that these six *PbCPP* genes exhibit relatively high expression levels under specific physiological processes and stress conditions. Therefore, the expression of the six PbCPPs under the three abiotic stresses was analyzed using quantitative reverse transcription polymerase chain reaction (qRT-PCR); the primer sequences are listed in Table S1. The stress treatments were as follows: polyethylene–glycol-induced drought–cold stress (10 °C) and salt stress (10% NaCl solution). Through analysis, it can be seen that the *PbCPP* genes are significantly up- or down-regulated under different stress conditions (Figure 8). Under drought stress (Figure 8), *PbCPP2*, *PbCPP3*, *PbCPP4*, *PbCPP8*, and *PbCPP10* were all significantly up-regulated. After PEG treatment, *PbCPP2* and *PbCPP4* peaked at 8 h and then decreased, whereas *PbCPP3* and *PbCPP8* peaked at 12 h and then decreased. *PbCPP10* began to decrease after 4 h. These findings indicate that the *PbCPP* gene family has strong drought tolerance. *PbCPP7* was down-regulated within 8 h of PEG treatment and then up-regulated to its peak, indicating that *PbCPP7* may be a key gene involved in the salt stress response. PbCPPs were significantly expressed when exposed to cold stress (Figure 8). Most *PbCPP* genes, especially *PbCPP*4, 7, 8, and 10, peaked at 2 h of cold stress and then began to decrease. *PbCPP*2 and *PbCPP*3 peaked at 12 h. These findings indicate that the *PbCPP* family has poor tolerance to cold stress. During exposure to salt stress (Figure 8), the expression levels of *PbCPP3*, *PbCPP4*, *PbCPP7,* and *PbCPP10* were the lowest at 2 h and then began to increase, while *PbCPP2* and *PbCPP*8 were slowly up-regulated. All genes except *PbCPP4* and *PbCPP10* peaked at 24 h, indicating that they had high salt tolerance and may play an important regulatory role in the long-term adaptation of plants to salt.

## 4. Discussion

Abiotic stresses are important factors affecting the growth and development of plants throughout their life cycle, and the *CPP* gene family plays a key role in the plant’s response to these stresses. Although *CPP* genes have been studied in many plants, there have been few studies of this gene family in the endangered woody plant *Phoebe bournei*. In this study, the *CPP* gene family was comprehensively analyzed in *Phoebe bournei*, revealing its significance in plant growth and development as well as adaptation to adversity.

The *CPP* gene family, namely the cysteine-rich polycomb-like protein (*CPP*) gene family, is a small family of transcription factors widely existing in plants and is involved in plant growth regulation and stress tolerance, such as drought, salt stress, and heat stress [37]. The number and characteristics of *CPP* gene family members vary among different plant species. For example, 8 *CPP* genes were identified in *Arabidopsis thaliana* [33], 11 in rice (*Oryza sativa*) [33], 20 in soybean (*Glycine max*) [40], 13 in maize (*Zea mays*) [8], 5 in cucumber (*Cucumis sativus*) [34], and 11 in tea (*Camellia sinensis*) [36], and they can have the same or different responses to different stresses. However, the *CPP* gene family in *Phoebe bournei* has not been studied and analyzed.

Our phylogenetic analysis showed that the *PbCPP* genes are divided into three subfamilies (A, B, and C), consistent with the classification in *Arabidopsis* and rice identified reported by Yang and Wang [31,33]. This conservation suggests that *PbCPP* genes may perform similar functions to those in other species. The identification of conserved motifs within PbCPPs indicates functional conservation across different subfamilies. The functions of their ancestors and the potential for conserved biological processes are highlighted [52,53]. *PbCPP* genes within the same subfamily are comparable in terms of structural features, such as exon–intron structure, conserved protein motif distribution, and conserved structural domains, further supporting the presumption of their functional similarity. For example, the distribution of conserved protein motifs is the same or basically the same in *PbCPP5* and *PbCPP7*; *PbCPP1*, *PbCPP6*, and *PbCPP8*; and *PbCPP4* and *PbCPP3*. Members of the same subfamily share the same motif composition and order, indicating that these genes may have similar functions. Changes in gene structure, including the number of exons and introns, may be the basis for the functional diversity observed in the *PbCPP* gene family, as Zhang observed in *soybeans*, where changes in gene structure are associated with diversity and adaptive changes in gene expression [40].

The chromosomal localization analysis showed that *PbCPP* genes are unevenly distributed across chromosomes, with some forming gene clusters. This clustering may be associated with tandem duplication events, which are known to contribute to the expansion and functional divergence of gene families in plants [54]. The discovery of fragment repeats involving *PbCPP* genes further confirms the role of repeats in the evolution of these genes. As Steven observed in *Arabidopsis*, the size of the gene family and the physical proximity of genes are closely related to the frequency of tandem repeat events. These events have a significant effect on the expansion and functional differentiation of the gene family [55].

The tissue-specific expression patterns of *PbCPP* genes suggest distinct roles in various developmental stages and tissues of *Phoebe bournei*. It is noteworthy that *PbCPP3* and *PbCPP4* were expressed at high levels in the roots and stems and low levels in the leaves, while most of the other genes were expressed at low levels in the roots, stems, and leaves of *Phoebe bournei*, especially in the leaves, showing obvious tissue specificity. This suggests that the functions of *PbCPP* genes may differ in different tissues and may be involved in regulating the growth and development of different tissues in plants. The differential expression of *PbCPP* genes under drought, salt, and cold stress conditions highlights their potential roles in stress response and adaptation. The up-regulation of certain *PbCPP* genes under these stress conditions implies their importance in plant defense mechanisms. Zhang and Song, for example, found similar expression patterns in plants such as maize under drought, salinity, and cold [3,8].

The analysis of cis-acting elements in the promoter regions of *PbCPP* genes revealed a wealth of regulatory motifs associated with hormone responses, stress signaling, light responses, and growth and development. The presence of these elements suggests that *PbCPP* genes are integrated into complex regulatory networks that modulate plant responses to environmental cues. The enrichment of light-responsive elements in PbCPP promoters is particularly noteworthy, because it suggests that these genes play a role in photomorphogenesis and in adapting to light stress, as described by Chattopadhyay [56]. The regulatory role of phytochromes in plant growth and development and the expression analysis of transcription factors regulated by blue light in *Arabidopsis thaliana* reported by Jiao proved that these transcription factors play an important role in the response of plants to light signals [57].

The identification of homologous genes and collinearity between *Phoebe bournei* and other plant species underscores the evolutionary conservation of the *CPP* gene family. This conservation likely reflects the fundamental roles of these genes in plant biology. The functional significance of the *PbCPP* gene family is further confirmed by the presence of conserved structural domains observed in *Arabidopsis* by Riechmann and the response of these genes to abiotic stresses [29].

When plants face drought stress, a decrease in stomatal conductance in leaves helps to reduce water loss and maintain the water status in cells, which is an important mechanism by which plants adapt to a water-deficit environment [58,59,60]. This study revealed that the promoter regions of PbCPP family members contain a large number of drought-responsive cis-acting elements, suggesting that the family may play a role in drought stress response. Through qRT-PCR analysis, we observed that the expression levels of most *PbCPP* genes were up-regulated under drought stress, which is similar to the expression pattern of the tomato *CPP* gene family under drought stress [37]. In addition, the expression of the *ZmCPP* gene in maize was up-regulated after 12 h of drought stress, which further confirmed the important role of the *CPP* gene family in plant drought resistance [8]. Under cold stress, the response pattern of the *PbCPP* gene family was similar to that of the *CPP* gene family in cucumber. Most of the *PbCPP* genes reached peak expression after 2 h of cold stress and then began to down-regulate, indicating that the PbCPP family has poor tolerance to cold stress. The expression levels of multiple *CPP* genes in *cucumber* changed upon cold stress, suggesting that the *CPP* gene family may have similar regulatory roles in the response of plants to cold stress [34]. Under salt stress, the expression pattern of the *PbCPP* gene family is similar to that of the *CPP* gene family in tomato. Most *PbCPP* genes still maintained high expression levels after 24 h of salt stress, indicating that they may play an important regulatory role in the long-term adaptation of plants to salt. Changes in the expression of multiple *CPP* genes in tomato under salt stress showed the complexity of the *CPP* gene family in the plant response to salt stress [37].

## 5. Conclusions

In this study, we identified 11 *PbCPP* genes in *P. bournei* and conducted a comprehensive analysis of their properties, relationships, structures, functions, and expression patterns. These 11 *PbCPP* gene family members are distributed across four chromosomes, with a wide variation in amino acid number. A phylogenetic analysis classified the *PbCPP* genes into three subfamilies: A, B, and C. A promoter cis-acting element analysis revealed that *PbCPP* genes contain diverse elements involved in responses to plant hormones, stress signals, and light and in growth and development. Notably, most *PbCPP* genes possess MYB binding sites that regulate drought-induced expression, suggesting their critical role in plant drought resistance. An expression analysis showed that *PbCPP3* and *PbCPP4* were highly expressed in the roots and stems but showed low expression in the leaves, while other genes exhibited limited expression in the roots, stems, and leaves. Additionally, a qRT-PCR analysis of six representative *PbCPP* genes under abiotic stress conditions, specifically drought, cold stress, and salt stress, revealed significant differences in their expression levels. These findings suggest that *PbCPP* genes play an essential role in stress responses. This study provides preliminary evidence of the *PbCPP* gene family’s involvement in various abiotic stress responses, offering an important foundation for understanding their roles in plant growth, development, and stress adaptation.

## Figures and Tables

**Table 1:** Detailed information on 11 *PbCPP* genes in *Phoebe bournei* and their encoded proteins.

Gene Name	Gene ID	AA/aa	MW/kDa	pI	II	AI	GRAVY	Subcellular Localization
OF11936-RA	PbCPP1	142	15,718.56	5.46	45.27	53.66	−0.81	Nucleus
OF04268-RA	PbCPP2	423	46,902.83	5.68	72.08	51.65	−0.799	Nucleus
OF21316-RA	PbCPP3	685	75,008.06	5.49	64.13	57.36	−0.822	Nucleus
OF27418-RA	PbCPP4	813	88,999.17	5.69	54.63	62.46	−0.699	Nucleus
OF13684-RA	PbCPP5	460	49,459.6	7.17	51.88	61.22	−0.61	Nucleus
OF11935-RA	PbCPP6	272	30,496.31	8.57	41.51	59.85	−0.681	Nucleus
OF22648-RA	PbCPP7	568	60,799.64	7.94	52.3	68.08	−0.445	Nucleus
OF27954-RA	PbCPP8	149	16,915.16	8.31	69.08	55.64	−0.721	Nucleus
OF00758-RA	PbCPP9	580	64,246.22	8.43	63.73	61.02	−0.696	Nucleus
OF00759-RA	PbCPP10	580	64,940.85	8.92	67.23	57.45	−0.864	Nucleus
OF04279-RA	PbCPP11	690	76,270.21	5.59	61.23	68.99	−0.586	Nucleus

Note: AA: number of amino acids; MW: molecular weight; pI: theoretical isoelectric point; II: instability index; AI: aliphatic index; GRAVY: grand average of hydropathicity.

## Data Availability

Data are contained within the article.

## Supplementary Materials

The following supporting information can be downloaded at: https://www.mdpi.com/article/10.3390/plants14050803/s1, Table S1 Primers used in qRT-PCR test.
